# Concentration and Temperature Effects on Water and Salt Permeabilities in Osmosis and Implications in Pressure-Retarded Osmosis

**DOI:** 10.3390/membranes8030039

**Published:** 2018-07-08

**Authors:** Edvard Sivertsen, Torleif Holt, Willy R. Thelin

**Affiliations:** SINTEF, Richard Birkelands veg 3, N-7034 Trondheim, Norway; torleif.holt@sintef.no (T.H.); willy.thelin@sintef.no (W.R.T.)

**Keywords:** osmosis, osmotic power, pressure-retarded osmosis, water permeability, salt permeability, temperature effect, concentration effect

## Abstract

Osmotic power extracted from the mixing of freshwater with seawater is a renewable energy resource that has gained increasing attention during recent years. The estimated energy can significantly contribute to the production of power worldwide. However, this power production will be subject to variation due to both local conditions and seasonal variation. The present paper explores the effect of concentration and temperature on water and salt fluxes in osmosis at zero transmembrane pressure for five different membranes. Further, the measured fluxes have been utilized to model water and salt permeabilities (*A* and *B*), and the structure parameter (*S*). The observed flux variations at different combinations of concentration and temperature have been ascribed to skin properties, i.e., changes in *A* and *B* of each membrane, whereas *S* was assumed constant within the range of concentrations and temperatures that were tested. Simplified equations for the variation in *A* and *B* with temperature and concentration have been developed, which enable *A* and *B* to be calculated at any concentration and temperature based on permeabilities determined from osmotic experiments at standard test conditions. The equations can be used to predict fluxes and specific power production with respect to geographical and seasonal variations in concentration and temperature for river water/seawater pressure-retarded osmosis. The obtained results are also useful for forward osmosis processes using seawater as draw solution.

## 1. Introduction

During recent years, osmotic power from mixing river water and seawater has gained increasing attention in the field of renewable energy research [1,2]. The latest estimate of worldwide power potential was reported to 1700 TWh per year [3].

The principle of osmotic power is to utilize the mixing energy when two solutions with different salinities are mixed. Pressure-retarded osmosis is one of the technologies that may be used to harvest this energy [2,4]. For typical conditions, the reversible mixing energy when 1 kg of freshwater is mixed with an excess of seawater is 2.7 kJ [4]. Since a pressure retarded osmosis (PRO) power plant typically will be operated at half the osmotic pressure difference between the two solutions, the maximum mixing energy that can be extracted will be limited to 50% of the reversible mixing energy. However, frictional losses in, e.g., membrane modules, piping and pumps will reduce the exploitable net energy, and it will be realistic to exploit only approximately 40% of the reversible mixing energy [5].

Local conditions such as seawater concentration and water temperature will affect the power that can be extracted per unit area of membrane, i.e., the specific power. In addition, the seasonal variation of concentration and temperature will affect power production and must be addressed when designing a PRO power plant. In this respect, it will be essential to estimate the water and salt fluxes through the membrane as functions of temperature and concentration in order to enable the prediction of power production at relevant conditions.

Other processes exploiting osmotic energy have recently been proposed, i.e., osmotic energy recovery to reduce energy consumption for the desalination of seawater by reverse osmosis [6,7,8,9,10,11,12,13]. In addition, several treatment processes exploiting the osmotic driving force have been proposed [14,15,16,17]. All concepts will depend on draw concentration and temperature.

Performance data published in the literature are typically measured using 3.5 wt % NaCl and/or 1 M NaCl, e.g., Han et al. [18]. However, data spanning concentrations in the range of 20–35 g/L that will be relevant for conditions inside a membrane element for river water/seawater PRO and osmotic energy recovery or treatment processes using forward osmosis (FO) with seawater as draw solution are, to our knowledge, missing. Further, most of the data presented in the literature are given at 20 °C, 25 °C, or at the less specific condition “room temperature”.

A few papers have addressed the effect of temperature on osmotic performance. Zhao and Zou [19] studied the effect of temperature on membrane performance in FO desalination and their findings confirmed that the flux increased with increasing temperature. A Cellulose Triacetate membrane (CTA) from Hydration Technology Innovations (HTI) operated in FO mode was used in the experiments with a 1.5 M Na_2_SO_4_ draw solution and brackish water as feed solution. The water flux was found to increase by 3.1% per degree Celsius when the temperature was increased from 25 °C to 35 °C, and by 1.2% per degree Celsius when the temperature was increased from 35 °C to 45 °C, indicating a non-linear effect.

She et al. [20] also used a CTA membrane from HTI and measured the water flux at 25 °C and 35 °C at various pressures, applying a 1 M NaCl draw solution and a 1 mM NaCl feed solution. At isobaric condition, the flux increased approximately 4.1% per temperature degree increase, whereas the specific power increased by 3.4% per degree when the temperature was increased from 25 °C to 35 °C. They related the temperature effect to increased water and salt permeabilities and noted that the ratio between water and salt permeabilities was close to constant. They also concluded that increased water permeability was the dominating factor to improved water flux. Further, the increase in diffusivity at elevated temperatures was claimed to reduce the internal concentration polarization in membrane support, which also contributed to the increased water flux.

Kim and Elimelech [21] studied the effect of temperature on the water flux by also using a CTA membrane from HTI. They measured the isobaric water flux at 20 °C and 30 °C with a 0.5 M NaCl feed solution and 1 M, 1.5 M, or 2 M NaCl draw solutions, respectively. The observed increase in water flux for the different draw concentrations was 7.1%, 3.9%, and 5.0% per degree increase in temperature, respectively. They related the increased water flux to increased water permeability and claimed that the simultaneous increase that was expected in salt permeability was not important for the efficiency in PRO. A comparison of the measured fluxes at different concentrations at constant temperature resulted in an increase in the water flux of approximately 3% per g/L increase in concentration difference across the membrane.

Touati et al. [22,23] also studied the effect of temperature and concentration on the water flux using a CTA membrane from HTI and a membrane from the Fraunhofer IGB Institute. Both the water and salt permeabilities were fitted to Arrhenius equations, giving good correlation to the observed temperature dependency. An increase of 0.33 W/m^2^ was reported for the CTA membrane when the temperature changed from 25 °C to 60 °C, equal to approximately 1% increase in specific power per degree increase in temperature.

The work presented in the current paper has focused on the effects of concentration and temperature on water and salt fluxes in FO/PRO. The water and salt fluxes have been measured in both FO and PRO mode at isobaric conditions for five membranes at different combinations of concentration and temperature. The main objective was to quantify the impact of concentration and temperature on water and salt fluxes, respectively, and to model these effects in terms of variation in water and salt permeabilities. As a result, simplified equations that can be used to predict the impact of variations in concentration and temperature on water and salt permeabilities for a given membrane have been developed. Utilization of these equations presupposes that the water and salt permeability of the membrane is obtained for one single combination of concentration and temperature, e.g., under standard test conditions. Subsequently, water and salt fluxes, as well as PRO performance, can be calculated for any process condition using an appropriate membrane transport model.

## 2. Theory

### 2.1. Transport Model

**Figure 1:**
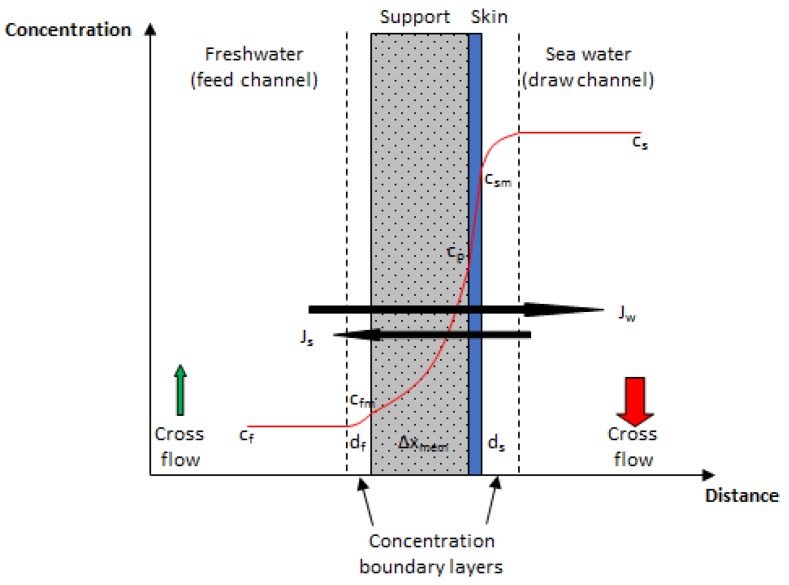
Concentration profile over a Thin Film Composite (TFC) membrane and the boundary layers in PRO, modified from [24].

In PRO, water will be transported against a pressure gradient due to the difference in osmotic pressure between the two water sources flowing on each side of the membrane. The net volume increase on the high saline side, which is operated at elevated pressure, can be converted into power in a turbine. Figure 1 illustrates the concentration profile over a Thin Film Composite (TFC) membrane in PRO [24], including concentration boundary layers on either side of the membrane, and indicates the direction of salt and water fluxes.

The produced power, *P*, equals the amount of water transported through the membrane multiplied with the hydraulic pressure difference, i.e.,

(1) $$P=J_{w}\Delta p$$

where *J_w_* is the water flux and Δ*p* is the pressure difference across the membrane. Since the water flux in PRO will decrease as the pressure difference increases, the produced specific power will have a theoretical optimum at half the osmotic pressure difference. Different model frameworks describing the transport of salt and water through the membrane have been developed by several authors [4,25,26,27,28,29]. For the work presented in the current paper, a transport model developed by Thorsen and Holt [4], which calculates the water and salt transport in four transport zones, i.e., the boundary layer on both membrane surfaces, inside the porous support structure, and across the membrane skin, was applied. The model will be briefly presented in the following section, whereas a more detailed deduction, including the solving of respective mass balances for the different transport zones is given in the Supplementary Materials.

The mass transport through the membrane skin can be described by the flux equations

(2) $$J_{w}=A(\Delta \pi_{skin}-\Delta p)$$

and

(3) $$J_{s}=-B\Delta c_{skin}=-B(c_{sm}-c_{p})$$

where *J_s_* is the salt flux, *A* is the water permeability, *B* is the salt permeability, and Δ*π_skin_* is the osmotic pressure that corresponds to the concentration difference of salt over the membrane skin. It can be shown that the concentration difference over the membrane skin can be expressed as

(4) $$\Delta c_{skin}=\frac{c_{s}-c_{f}e^{\left(\frac{(S+d_{s}+d_{f})J_{w}}{D}\right)}}{e^{\left(\frac{d_{s}J_{w}}{D}\right)}+\frac{B}{J_{w}}\left[e^{\left(\frac{(S+d_{s}+d_{f})J_{w}}{D}\right)}-1\right]}$$

where the structure parameter *S*, which represents the effective diffusion length through the support membrane, has been introduced (cf. Equation (S5)). This equation relates the salt concentration difference over the membrane skin to both the bulk concentration and the boundary layer thickness on both sides of the membrane, as well as the characteristic membrane parameters. Hence, the model describing the osmotic mass transport through a PRO membrane includes five parameters, where *A*, *B* and *S* describe the membrane characteristics, and *d_s_* and *d_f_*, which describe the thickness of the respective boundary layers, represent the flow regimes on each side of the membrane.

### 2.2. Impact of Temperature and Concentration on PRO Performance

It can be seen from the Van’t Hoff relationship in Equation (5) that both the temperature and concentration will determine the osmotic pressure, and thus the PRO performance.

(5) π=iRTc

*R* is the ideal gas constant, *T* is the absolute temperature, and *i* reflects the deviation from the ideal solution. The latter has been determined to 1.9 for NaCl by using linear regression and literature data for the osmotic pressure [30].

In addition to the impact of temperature and concentration that is given by the osmotic pressure term, membrane parameters *A* and *B* can also be influenced by variation in temperature and concentration. The working hypothesis of this paper is based on the assumption that the temperature and concentration relationship for the water and salt permeabilities will follow the same relationship as diffusion coefficients in liquids [31]. Following this analogy, the water and salt permeabilities can be expressed by:

(6) $$\frac{A}{A_{0}}\;=\beta_{mem}^{A}\frac{T}{T_{0}}\frac{\mu_{0}}{\mu }$$

and

(7) $$\frac{B}{B_{0}}\;=\beta_{mem}^{B}\frac{T}{T_{0}}\frac{\mu_{0}}{\mu }$$

*A*_0_ and *B*_0_ correspond to water and salt permeabilities measured at reference conditions *T*_0_ and *c*_0_, respectively. The *β* coefficients reflect the temperature and concentration dependencies of the water and salt permeabilities that are not related to the suggested temperature and water viscosity relationship. These coefficients must be determined experimentally. A more detailed discussion of the impact of temperature and concentration on the permeabilities are given in the Supplementary Materials.

## 3. Materials and Methods

### 3.1. Apparatus

**Figure 2:**
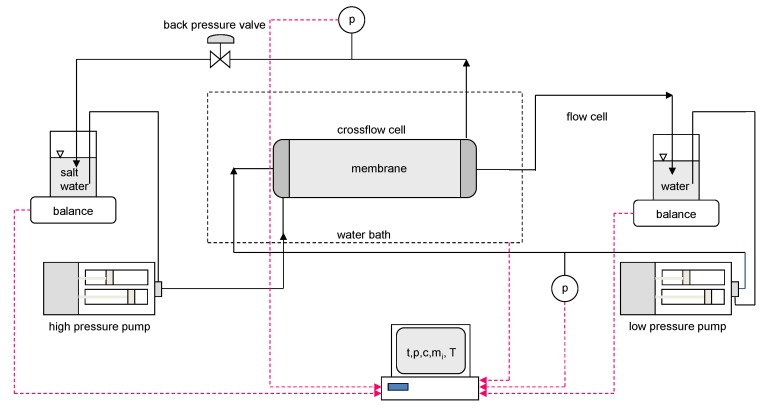
Simplified flow diagram for the two cross flow apparatuses used in the study.

Two cross-flow cells with effective membrane areas of 6.1 cm^2^ (1.1 cm × 5.5 cm) and 9.5 cm^2^ (1.1 cm × 8.6 cm), respectively, have been used in this study. Figure 2 shows a simplified flow diagram for the two cross-flow apparatuses. Water was fed to each side of the membrane by using dual-piston pumps with displacement volumes of approximately 10 mL/stroke. The feed reservoirs were placed on balances, and the discharge from the membrane cell was recycled back to the reservoirs.

The membrane cells and cooling/heating coils upstream of the membrane cells were immersed in a water bath to control the temperature during the experiments. The pressure on both sides of the membrane, the temperature in the water bath, and the readings of the balances were monitored and logged in a data file at regular intervals.

### 3.2. Standard Test Protocol

As prescribed by the manufacturer, three of the membranes were immersed in 50 vol % methanol for 60 s and subsequently immersed in rinsed water for a minimum of 60 min prior to assembly in the membrane cells. The membranes that were not preconditioned with methanol were immersed in distilled water prior to assembly in the membrane cells.

Two pieces of a permeate spacer of 0.5 mm thickness were applied in the freshwater channel comprising a channel thickness of 1.0 mm, and a diamond spacer of 0.7 mm thickness was applied in the saltwater channel.

After assembly, a hydraulic water permeability test was performed using degassed, rinsed water at 20 °C, and equal flow rates on both sides of the membrane (60 mL/h). The water flux was measured for minimum 60 min at 5–7 different pressures, ranging from 1–10 bar.

After the hydraulic water permeability test, two independent osmotic flow experiments were performed at 20 °C and isobaric conditions, one in PRO mode, i.e., draw solution against the membrane skin, and one in FO mode, i.e., draw solution against the membrane support. The saltwater was made from NaCl (p.a.) and degassed and rinsed water. Degassed and rinsed water was also used as feed water on the low concentration side. Equal flow rates were used for both pumps (300 mL/h). The water flux was determined based on weight changes in both reservoirs. The reported water fluxes were estimated for the initial phase of the experiments, i.e., the first two hours, before the dilution of saltwater and salt accumulation in the freshwater influenced the experiment. Salt fluxes were determined by potentiometric analyses of Cl^−^ ions in the freshwater reservoir at the end of the experiments.

### 3.3. Membranes

Five diverse types of noncommercial proprietary FO/PRO membranes were used in the study. One of the tested membranes was an asymmetric CTA membrane, whereas the four remaining membranes were TFC membranes referred to as TFC1–TFC4. Water permeability was in the 2 × 10^−12^–3 × 10^−11^ m/s/Pa range, salt permeability was in the 9 × 10^−8^–2 × 10^−6^ m/s range, whereas the structure parameter was in the 0.2–2.2 mm range.

### 3.4. Experimental Design

To systematically study the impact of concentration and temperature on osmotic flux, several experiments were performed at different temperatures and concentrations, varying around the standard test conditions, which are 20 °C and 28 g/L NaCl. Concentration and temperature have been varied according to two alternative designs, (1) a Central Composite Design (CCD); or (2) a face-centered Central Composite Design (face-centered CCD) which are described in more detail in the Supplementary Materials. Two osmotic flow experiments were performed for each combination of temperature and concentration, one in FO mode and one in PRO mode. The two osmotic flow experiments performed for each test condition resulted in four fluxes, two salt fluxes and two water fluxes. In total, 118 water fluxes with 118 corresponding salt fluxes were measured.

## 4. Results and Discussion

### 4.1. Measured Water and Salt Fluxes

**Figure 3:**
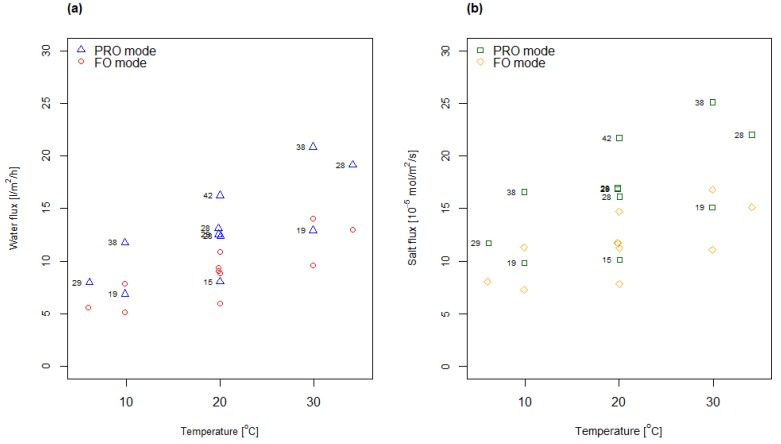
Water (**a**) and salt (**b**) fluxes measured for the CTA membrane at different concentrations and temperatures according to a CCD. The figures indicated for each data point (PRO mode only) correspond to the applied draw concentration (g/L NaCl) in that experiment.

Figure 3 shows measured water and salt fluxes as a function of temperature for the CTA membrane. As can be observed, the water and salt fluxes measured in FO mode are lower compared to the fluxes measured in PRO mode at the same conditions, which can be explained by higher internal concentration polarization in membrane support when the draw solution faces the support side of the membrane. This is in accordance with observations by other researchers [4,32,33].

The overall observed trend indicates that both water and salt fluxes increase with temperature. This was expected and in accordance with other results reported in the literature [20,21]. The effect of the concentration is implicitly given in Figure 3, e.g., by studying the water fluxes measured in PRO mode at 20 °C the water flux increases as the concentration increases from 15 g/L via 28 g/L to 42 g/L salt. Evaluation of all flux data confirmed a similar relationship, indicating that both salt flux and water flux were increasing with increasing concentration for all temperatures tested.

### 4.2. Analysis of Variance of Flux Data

**Figure 4:**
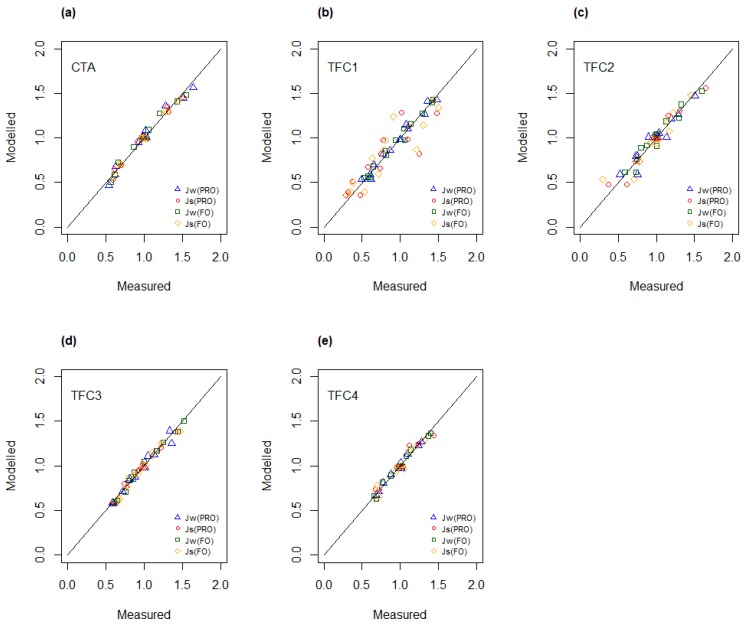
Linear regression modelled versus measured water and salt fluxes for (**a**) CTA; (**b**) TFC1; (**c**) TFC2; (**d**) TFC3 and (**e**) TFC4.

To obtain an objective measure of the observed effects of temperature and concentration on measured fluxes, the experimental data have been analyzed by using Analysis of Variance (ANOVA). It was found that the main effects of both temperature and concentration were significant for both water and salt fluxes (Table S2). This observation applied to all membranes. The interaction effect between temperature and concentration was observed to have minimal impact and was found to be significant for only three salt fluxes. In general, the high values of the adjusted coefficient of determination, R^2^(adj) (Table S2), indicate that the resulting regression models for water and salt flux give an excellent representation of experimental data sets that were obtained from the osmotic experiments performed with each membrane. In Figure 4, the modelled values for water and salt flux are plotted as function of experimental data, i.e., measured fluxes. Most data points appear on a line with a slope of unity, indicating a good fit between modelled values and measured data.

### 4.3. Determination of A, B and S as Function of Concentration and Temperature

Assuming constant thickness of boundary layers on membrane surfaces, Equation (4) will comprise three unknown parameters, i.e., water permeability, salt permeability, and structure parameter. Since water and salt fluxes have been measured in both FO and PRO mode, the degrees of freedom are sufficient to enable determination of the three parameters in each test condition. The assumption of constant boundary layer thickness is further discussed in the Supplementary Materials.

**Figure 5:**
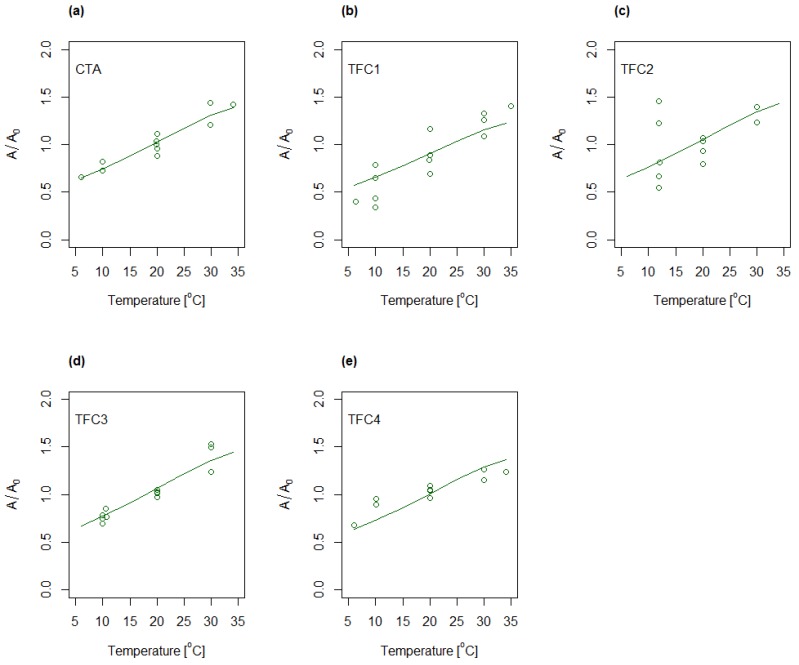
Relative changes in water permeability as function of temperature for (**a**) CTA; (**b**) TFC1; (**c**) TFC2; (**d**) TFC3 and (**e**) TFC4. Concentration dependency is implicitly shown for each temperature by multiple data points representing different concentrations. Solid lines represent the regression models following Equation (6).

**Figure 6:**
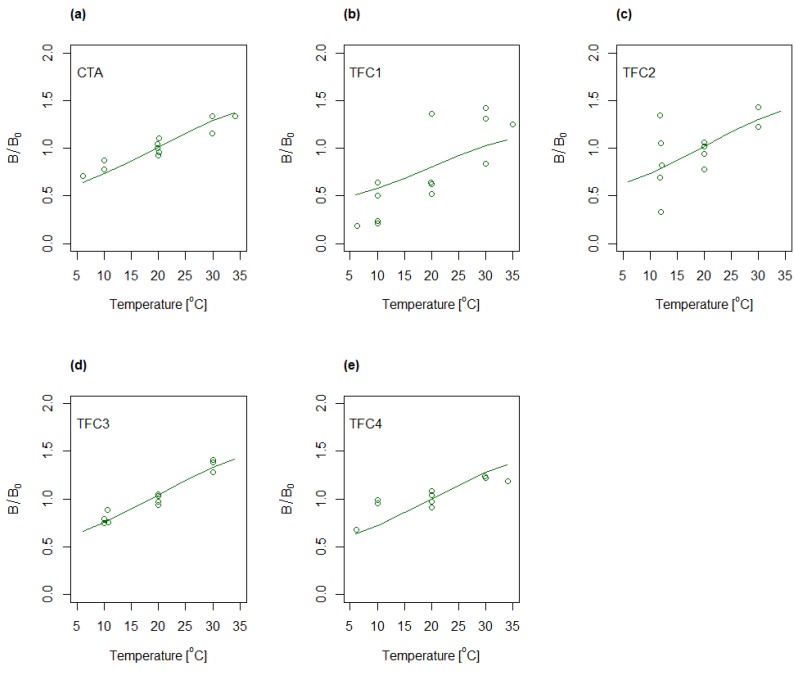
Relative changes in salt permeability as function of temperature for (**a**) CTA; (**b**) TFC1; (**c**) TFC2; (**d**) TFC3 and (**e**) TFC4. Concentration dependency is implicitly shown for each temperature by multiple data points representing different concentrations. Solid lines represent the regression models following Equation (7).

The determination of characteristic membrane parameters followed a two-step process. Firstly, *A*, *B* and *S* were determined freely for each temperature and concentration combination, and secondly, *A* and *B* were remodeled by presuming constant structure parameter equal to the average values determined for the various conditions in the first step. The resulting water and salt permeabilities for the five membranes when presuming constant structure parameter are shown in Figure 5 and Figure 6, respectively. The permeabilities are shown relative to the values determined for the center point condition (28 g/L and 20 °C). Both water and salt permeabilities were observed to increase with increasing temperature.

### 4.4. Modelling of A and B

The experiments were also modelled by applying Equations (6) and (7) and subsequently analyzed by ANOVA (Table S4). The predicted values obtained by using the mentioned regression models are shown in Figure 5 and Figure 6 as solid lines for varying temperature and constant concentration (28 g/L). Evaluation of the calculated R^2^(adj) (cf. Supplementary Materials) indicates a good fit to the water permeability model for four of the membranes, CTA, TFC1, TFC3 and TFC4, whereas TFC2 deviates somewhat from the model. Further, the models for salt permeability for TFC1 and, to some degree, TFC2, were observed to have relatively low R^2^(adj), indicating a less good fit between experimental data and modelled values. The latter was partly ascribed to the uncertainty in the salt fluxes.

An interesting finding from the data analysis was that the regression coefficients found for the water and salt permeability when using Equations (6) and (7), respectively, were close to unity. Presuming regression coefficients equal to unity, the water and salt permeabilities can be estimated at any concentration and temperature if the three characteristic membrane parameters are determined experimentally for one combination of concentration and temperature, e.g., at the condition corresponding to the center point (28 g/L and 20 °C). This presumption implies that the water and salt permeabilities can be calculated from the following simplified equations

(8) $$A\;=A_{0}\frac{T}{T_{0}}\frac{\mu_{0}}{\mu }$$

and

(9) $$B\;=B_{0}\frac{T}{T_{0}}\frac{\mu_{0}}{\mu }$$

respectively.

**Figure 7:**
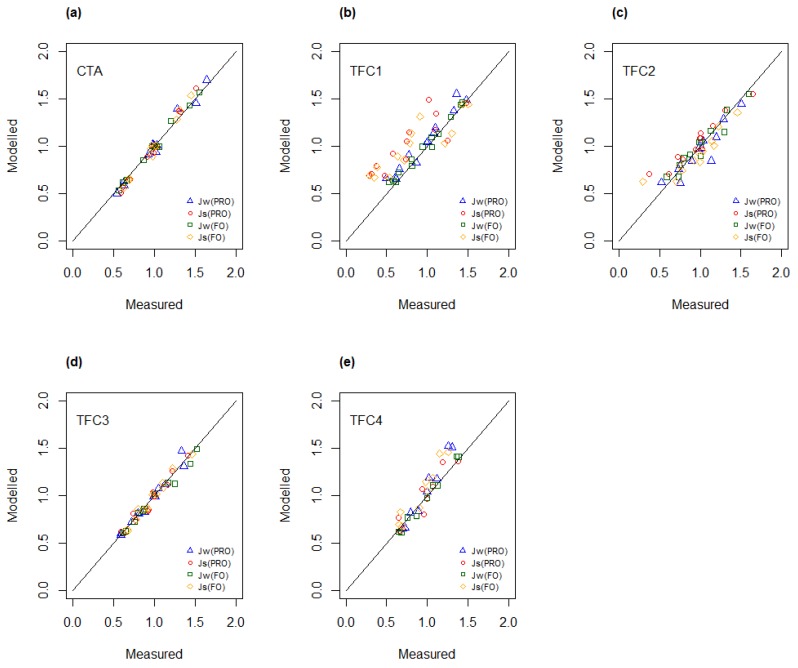
Modelled water and salt fluxes using water and salt permeabilities from Equations (8) and (9), respectively, as function of measured fluxes for (**a**) CTA; (**b**) TFC1; (**c**) TFC2; (**d**) TFC3 and (**e**) TFC4.

To test this assumption, the water and salt fluxes were calculated for the different test conditions by applying Equations (8) and (9) and compared with corresponding experimental data. *A*_0_ and *B*_0_ refer to the standard test condition (28 g/L and 20 °C). Figure 7 shows modelled versus measured fluxes for the five different membranes. It can be observed that the correlation between measured and modelled fluxes was generally high, with some deviation for the TFC1 membrane.

## 5. Implications in River Water/Seawater PRO

**Figure 8:**
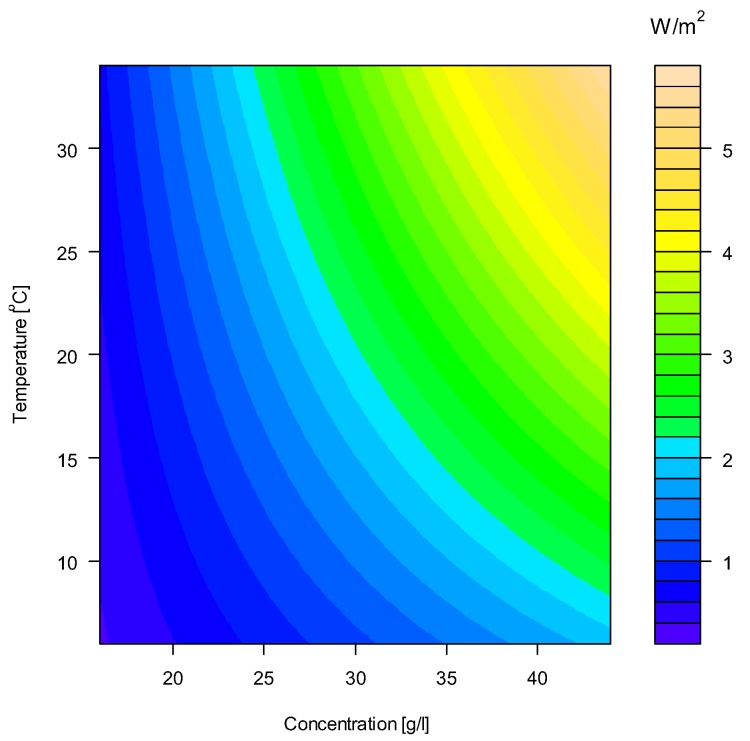
Contour plot of the specific power (W/m^2^) as function of concentration and temperature for the CTA membrane. Note that the operating pressure has been optimized for each combination of concentration and temperature.

The changes in water and salt permeabilities related to variation in operating conditions will directly influence the water flux and hence the power production. Local conditions at different sites and seasonal variations will determine the PRO potential and must be addressed in the planning phase of a PRO power plant. Using the CTA membrane as an example, the membrane parameters determined for each combination of concentration and temperature can be used to simulate the optimal power production, by optimizing the operating pressure for each condition. The simulated values of specific power at each combination of concentration and temperature have been used to construct a contour plot for specific power shown in Figure 8. The CTA membrane was determined to have an optimum performance of approximately 2 W/m^2^ at the center point, i.e., 28 g/L, 20 °C, and a significant dependency to both concentration and temperature was observed.

Norwegian rivers will typically have seasonal variations in temperature between 5 °C to 15 °C, whereas the temperature in the sea, which will additionally depend on intake depth, can be assumed to vary, in the range of 5 °C to 10 °C. Considering a seawater concentration of 3.5%, which is equivalent to 32 g/L NaCl (with respect to osmotic pressure), the expected variation in produced power will approximately be 30% within indicated temperature range. This corresponds to a 5% increase per degree Celsius. The power production of TFC membranes will vary in a comparable manner with respect to changes in temperature. The modelled impact on power due to variation in temperature corresponds well with values reported in the literature. Kim and Elimelech [21] have modelled the effect in PRO and estimated a 4.6% increase per °C, whereas She et al. [20] have measured the increase in specific power to 3.4% per °C.

## 6. Conclusions

The effect of concentration and temperature on water and salt fluxes in PRO has been investigated for five different membranes. The fluxes were measured at different combinations of concentration and temperature, and the effect of each variable was quantified. Further, the measured fluxes were used to model the membrane parameters *A*, *B* and *S* by fitting the PRO transport model to the measurements. It has been substantiated that the structure parameter can be considered independent of variations in concentration and temperature, and consequently that all experimental variation in the conducted FO/PRO experiments can be ascribed to changes in water and salt permeabilities.

The subsequent data analysis showed that the variation in the water and salt permeabilities could be modelled with reasonable accuracy by simply applying the dependency between absolute temperature and water viscosity. Therefore, the water and salt permeabilities can be estimated at any concentration and temperature by using two simplified equations that require water and salt permeabilities obtained for one single combination of concentration and temperature as input. In practice, it will be sufficient to perform two osmotic experiments, one in FO mode and one in PRO mode. The calculated fluxes that were found by using the simplified equations give a satisfactory correlation to experimental data. Thus, these equations can be considered a valuable tool for prediction of the impact of changes in concentration and temperature on the salt and water fluxes, and hence, the process efficiency.

The simplified equations are valid for saltwater concentrations corresponding to the 16–46 g/L NaCl range, and temperatures in the range of 6–36 °C, which covers most of the concentration and temperature ranges of interest utilizing seawater as a draw solution.

## Supplementary Materials

The following are available online at http://www.mdpi.com/2077-0375/8/3/39/s1, Details about the transport model development, assumptions and hypothesis of the impact of temperature and concentration on the film thicknesses and membrane parameters, presentation of the design of experiments used in the study, supplementary comments to the analysis of variance of the results, visualization of test conditions (Figure S1), measured water and salt fluxes (Figures S2–S5), relative changes in film thickness as function of temperature (Figure S6), relative changes in the structure parameter as function of temperature and concentration (Figure S7), summary of type of design and number of experiments for each membrane (Table S1), significant regression coefficients and coefficient of determination of the water and salt fluxes (Table S2), ANOVA of the structure parameter (Table S3), regression coefficients and coefficient of determination of the water and salt permeabilities (Table S4).
