# Supplementary material for: Concentration and Temperature Effects on Water and Salt Permeabilities in Osmosis and Implications in Pressure-Retarded Osmosis
Source: Membranes (Basel). 2018 Jul 8;8(3):39. doi: 10.3390/membranes8030039 (PMC6161017; doi:10.3390/membranes8030039)
Supplement: Supplementary file 1 [file membranes-08-00039-s001.pdf]

membranes

Supplementary Materials: Concentration and temperature effects on water and salt permeabilities in osmosis and implications in pressure retarded osmosis

Edvard Sivertsen, Torleif Holt and Willy R. Thelin

SINTEF, Richard Birkelands veg 3, N-7034 Trondheim, Norway; torleif.holt@sintef.no (T.H.); willy.thelin@sintef.no (W.R.T.)
* Correspondence: edvard.sivertsen@sintef.no; Tel.: +47-486-05-179

1. Theory

1.1 Transport Model

This section gives a detailed derivation of the transport model briefly presented in Thorsen and Holt [1]. The water flux in PRO is defined to be positive in the direction from the feed side to the draw side, whereas the salt flux is defined to be negative in the same direction. The mass transport of salt in the membrane support, and in each of the boundary layers, will equal the sum of the convective and diffusive salt transport due to the gradient in salt concentration. This can be expressed as

$$-J_s = D\frac{\varphi}{\tau}\frac{dc}{dx} - J_w c \tag{S1}$$

where J_s is the salt flux, J_w is the water flux, c is the salt concentration, D is the diffusion coefficient, φ is the porosity, and τ is the tortuosity of the support membrane. The negative value of the salt flux on the left side of the equation indicates that the salt flux is oppositely directed compared to the increasing distance x as defined in Figure 1 in the manuscript. Equation (S1) can be rearranged to

$$-\frac{J_s}{J_w} = \frac{D\varphi}{J_w\tau}\frac{dc}{dx} - c \tag{S2}$$

Further, Equation (S2) can be rearranged to an ordinary differential equation which can be integrated over the porous membrane support layer using the concentrations and distances defined in Figure 1, *i.e.* from $x = 0$ and $c = c_{fm}$ to $x = \Delta x_{mem}$ and $c = c_p$;

$$\int_{c_{fm}}^{c_p} \frac{dc}{\left(c - \frac{J_s}{J_w}\right)} = \int_{x=0}^{x=\Delta x_{mem}} \frac{dx}{\frac{D\varphi}{J_w\tau}} \tag{S3}$$

After integration of Equation (S3), applying the boundaries indicated in the above paragraph, the following expression can be obtained

$$ln\left[\frac{J_s + J_w c_p}{J_s + J_w c_{fm}}\right] = \frac{\tau\Delta x_{mem}}{\varphi}\frac{J_w}{D} \tag{S4}$$

describing the implicit relation between the water and salt flux in the membrane support.

The structure parameter, S, of the membrane support is defined as

$$S = \frac{\tau}{\phi}\Delta x_{mem} \tag{S5}$$

and resembles the effective diffusion length through the membrane support layer [1]. A similar integration of Equation (S3) over the boundary layers on each side of the membrane, gives

$$ln\left[\frac{J_s + J_w c_s}{J_s + J_w c_{sm}}\right] = \frac{d_s J_w}{D} \tag{S6}$$

and

$$ln\left[\frac{J_s + J_w c_{fm}}{J_s + J_w c_f}\right] = \frac{d_f J_w}{D} \tag{S7}$$

respectively. The porosity and tortuosity in the boundary layers will equal unity (*i.e.* φ = 1 and τ = 1) and d_s and d_f have been introduced as the thickness of the boundary layer on the skin and support side of the membrane, respectively.

The mass transport through the membrane skin is described by the flux equations

$$J_w = A(\Delta\pi_{skin} - \Delta p) \tag{S8}$$

and

$$J_s = -B\Delta c_{skin} = -B(c_{sm} - c_p) \tag{S9}$$

where $\Delta\pi_{skin}$ is the osmotic pressure that corresponds to the concentration difference of salt over the membrane skin. Combining the expression for the salt flux in Equation (S9) with the Equations (S4), (S6) and (S7), an expression for the concentration difference over the skin can be found

$$\Delta c_{skin} = \frac{c_s - c_f e^{\left(\frac{(S+d_s+d_f)J_w}{D}\right)}}{e^{\left(\frac{d_s J_w}{D}\right)} + \frac{B}{J_w}\left[e^{\left(\frac{(S+d_s+d_f)J_w}{D}\right)} - 1\right]} \tag{S10}$$

This equation relates the salt concentration difference over the membrane skin to the bulk concentration and the boundary layer thickness on both sides of the membrane as well as the characteristic membrane parameters *A*, *B* and *S*. *A* is included implicitly through the water flux J_w.

1.2 Impact of Temperature and Concentration on Water and Salt Permeabilities

It is reported in literature that both water and salt permeabilities will be temperature dependent [2-4], due to changes in the diffusion resistance in the membrane skin, and possibly also with concentration [4]. Changes in the diffusion resistance in the membrane skin may be caused by several phenomena occurring in the dense structure relating both to the kinetic properties of the diffusing species and to structural damping effects of the polymer. Different approaches may be pursued in order to explain mentioned effects in the diffusion coefficient [5]. Initially, we have chosen to use a temperature and concentration relationship similar to the Stoke-Einstein relationship describing the diffusion of large molecules in a solvent composed by small molecules. The Stoke-Einstein relationship is given as [5]

$$D = \frac{k_B T}{6\pi\mu R_0} \tag{S11}$$

where k_B is the Boltzmann constant, R_0 the diameter of the specie, μ is the viscosity of the solvent and *T* is absolute temperature. It follows from this equation that the diffusion coefficient will increase

with increasing temperature through the kinetic part of the equation which reflects the molecular movements. On the other hand, the diffusion will decrease with increasing viscosity of the fluid, which may depend on both temperature and concentration. Our hypothesis is that a similar dependency with respect to temperature and viscosity (meaning a damping effect) is experienced for both the water and the salt permeabilities. The temperature and viscosity relationship to the diffusion coefficient in Equation (S11) are therefore applied to study the relative changes in water and salt permeability, which gives the following expressions for the temperature and concentration dependency of water and salt permeabilities:

$$\frac{A}{A_0} = \beta_{mem}^{A} \frac{T}{T_0} \frac{\mu_0}{\mu} \tag{S12}$$

and

$$\frac{B}{B_0} = \beta_{mem}^{B} \frac{T}{T_0} \frac{\mu_0}{\mu} \tag{S13}$$

respectively. In these equations the A_0 and B_0 are the water and salt permeabilities measured at a reference temperature T_0 and reference concentration c_0. The β's are coefficients that include any temperature and concentration dependencies of the respective permeabilities that deviate from the expected relationship given in Equation (S12) and (S13).

Following the analogy with diffusion coefficients in solutions, the changes in permeabilities can be ascribed to molecular movements reflected in the temperature-part of the equation, and additionally to a damping factor for the transport of salt and water through the membrane skin. The water viscosity was used as a first approximation for the latter. Together with the direct temperature relationship the proposed relationships proved to explain the observed behavior well (cf. Section 3 and results section of the main paper).

1.3 Impact of Temperature and Concentration on Structure Parameter and Film Thickness

The structure parameter is defined in Equation (S5) and depends on the thickness, the porosity and the tortuosity of the membrane support. For a limited range of variation in temperature and salt concentration these properties can be assumed to be constant provided that the thermal expansion is insignificant and that the polymer do not swell or shrink due to variations in the salt concentration. Consequently, the structure parameter is expected to be independent of variations in salt concentration and temperature within the experimental region explored in this study.

Further, the mass transfer resistance in the boundary layers on the surfaces of the membrane will also contribute to the overall mass transfer resistance according to Equation (S10). It has been demonstrated [1,6] that the resistance in the boundary layer on the membrane support in most cases will be negligible compared to the resistance exerted by the boundary layer on the membrane skin.

The boundary layer thickness can be expressed with a mass transfer correlation on the form [5]

$$Sh = a\ \mathrm{Re}^{b} Sc^{c} \tag{S14}$$

where the Sherwood number is $Sh = kd_h/D = d_h/d$, the Reynolds number is $Re = \rho v d_h/\mu$, the Schmidt number is $Sc = \mu/\rho/D$ and a, b and c are empirical coefficients. Different correlations exist depending on flow regime and application, and the expression

$$Sh = 0.2\ \mathrm{Re}^{0.57} Sc^{0.40} \tag{S15}$$

has currently been reported for use in PRO at low Reynolds numbers and spacer filled channels [7]. To demonstrate the effect of concentration and temperature on the film thickness Equation (S15) has been rearranged to:

$$d = e \ \mathrm{Re}^{-b} Sc^{-c} \quad \text{(S16)}$$

where *e* is a new empirical coefficient. Equation (S16) shows that a change in concentration or temperature will influence the boundary layer thickness through changes in the Reynolds number and the Schmidt number, i.e. through changes in density, viscosity and in the diffusion coefficient.

2. Materials and Methods

In order to study the impact of concentration and temperature on water and salt fluxes systematically a Design of Experiment (DoE) strategy [8] have been applied, where the concentration and temperature have been varied either according to a central composite design (CCD) or a face-centred central composite design (face-centred CCD). The latter design was employed for some membranes due to insufficient cooling water supply during the warm summer months where the lowest temperature condition was not achieved, thus disregarded the use of central composite design for those experiments. Both designs are illustrated in Figure S1, which additionally defines the experimental concentration and temperature region.

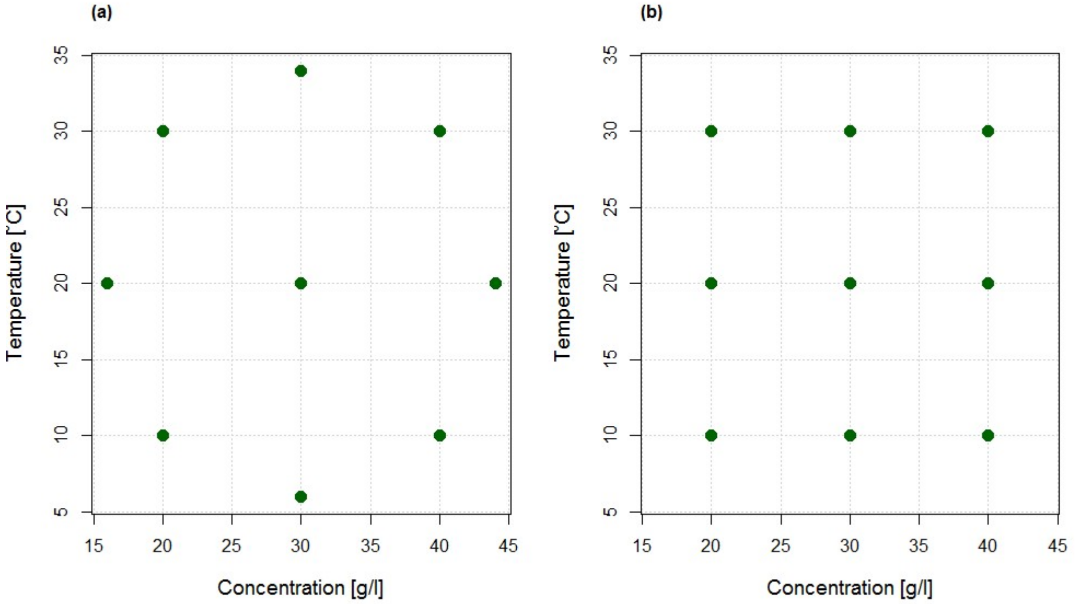

Figure S1. Experimental design indicating test conditions; (**a**) central composite design (CCD), (**b**) face centred central composite design (face-centred CCD).

Table S1 indicates type of design and the number of experiments performed for each membrane.

To obtain an objective measure of the effects of temperature and concentration on the measured fluxes, the data have been analyzed by analysis of variance (ANOVA) utilizing the inherent advantages of the experimental design. The following linear model was fitted to the experimental flux data

$$\text{response} = \beta_0 + \beta_t t + \beta_c c + \beta_{tc} tc \quad \text{(S17)}$$

The β's are regression coefficients describing the effect of the respective variables. It should be noted that the experimental data contain sufficient degrees of freedom to obtain good estimates of

the regression coefficients in the ANOVA, and their significance were tested using t-statistics. In this study a significance level of 5 % was used, i.e. the p-value of the variables obtained in the ANOVA should be less than 0.05 before the effect of the variable was considered significant.

Table S1. Maximum applied pressure in the permeability test, and number of experiments performed with the different membranes.

Membrane	Maximum Trans Membrane Pressure during Permeability Test [bar]	Design	Number of Temperature-Concentration Combinations
CTA	9	CDD	9 + 2 replicates
TFC1	10	CDD	11 + 2 replicates
TFC2	10	Face-centred CCD	11 + 1 replicate
TFC3	10	Face-centred CCD	9 + 3 replicates
TFC4	5	CDD	9 + 2 replicates

3. Results

3.1. Analysis of Variance of Flux Data

The measured water and salt fluxes as function of temperature for the TFC membranes are shown in Figure S2 through Figure S5, respectively. The corresponding measured fluxes for the CA membrane are shown in Figure 3 in the main paper. The effect of the concentration is also implicitly shown, where the figures indicated for each data point (PRO mode only) give the applied draw concentration. For further discussion see the main paper.

The results from ANOVA of flux data from the osmotic experiments are shown in Table S2. It can be observed that the effects of both temperature and concentration were found to be significant for both water and salt fluxes for all membranes. However, the interaction effect of temperature and concentration was observed to have low impact and was found to be significant for only three salt fluxes and none of the water fluxes. It should be noted that the regression model for the salt flux of the TFC1 membrane has a significantly lower R^2(adj), indicating less good fit to the data set. This can likely be ascribed to the uncertainty (standard deviation) in the salt analyses.

Table S2. Regression coefficients and R^2(adj) for relative water and salt fluxes measured in PRO mode and FO mode. Note that only coefficients having a p-value less than 0.05 are shown.

		PRO					FO				
		β_0	β_c	β_t	β_{ct}	R^2(adj)	β_0	β_c	β_t	β_{ct}	R^2(adj)
J_w	CTA	-0.317	0.025	0.031		0.966	-0.175	0.020	0.030		0.971
	TFC1	-0.069	0.017	0.029		0.973	0.014	0.013	0.030		0.980
	TFC2	-0.127	0.021	0.026		0.896	-0.081	0.015	0.034		0.931
	TFC3	0.023	0.014	0.027		0.959	0.018	0.012	0.034		0.980
	TFC4	0.252	0.012	0.019		0.978	0.122	0.014	0.024		0.975
J_s	CTA	0.117	0.015	0.007	0.0005	0.989	0.158	0.014	0.011	0.0004	0.992
	TFC1	-0.246	0.015	0.031		0.711	-0.171	0.010	0.037		0.773
	TFC2	-0.400	0.026	0.032		0.944	-0.215	0.020	0.030		0.864
	TFC3	0.152	0.013	0.013	0.0003	0.988	0.112	0.014	0.024		0.961
	TFC4	0.093	0.019	0.019		0.911	0.200	0.015	0.018		0.931

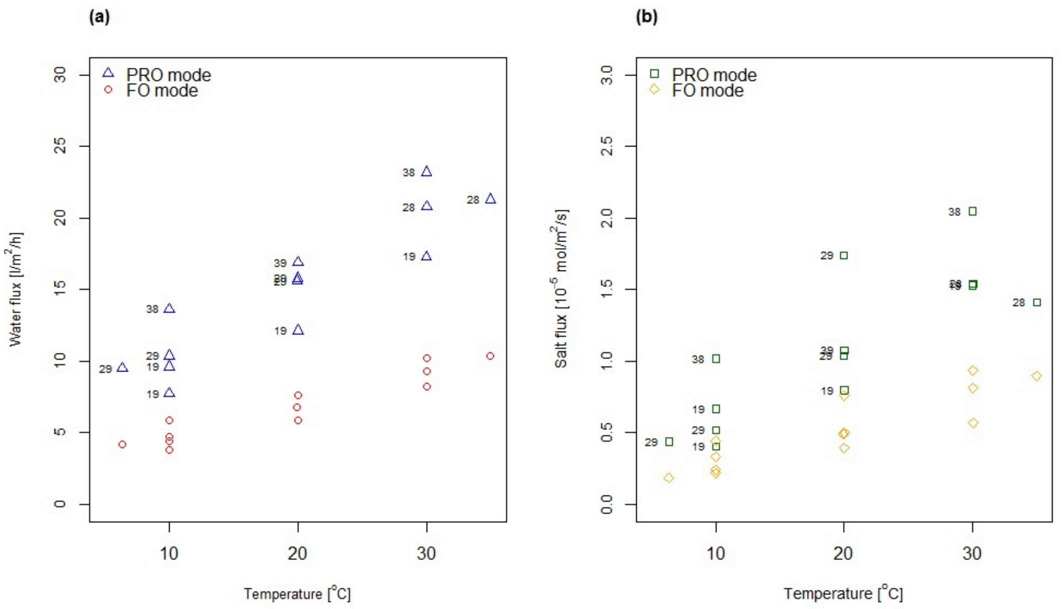

Figure S2. Water (**a**) and salt (**b**) fluxes measured for the TFC1 membrane at different concentrations and temperatures according to a CCD. The figures indicated for each data point (PRO mode only) correspond to the applied draw concentration (g/l NaCl) in that experiment.

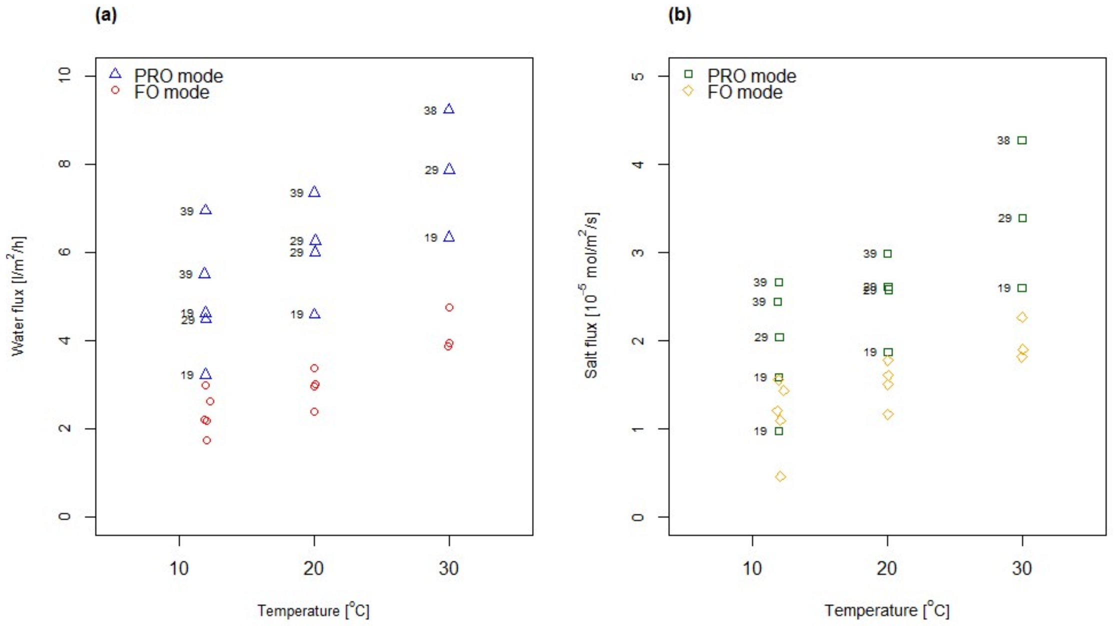

Figure S3. Water (**a**) and salt (**b**) fluxes measured for the TFC2 membrane at different concentrations and temperatures according to a CCD. The figures indicated for each data point (PRO mode only) correspond to the applied draw concentration (g/l NaCl) in that experiment.

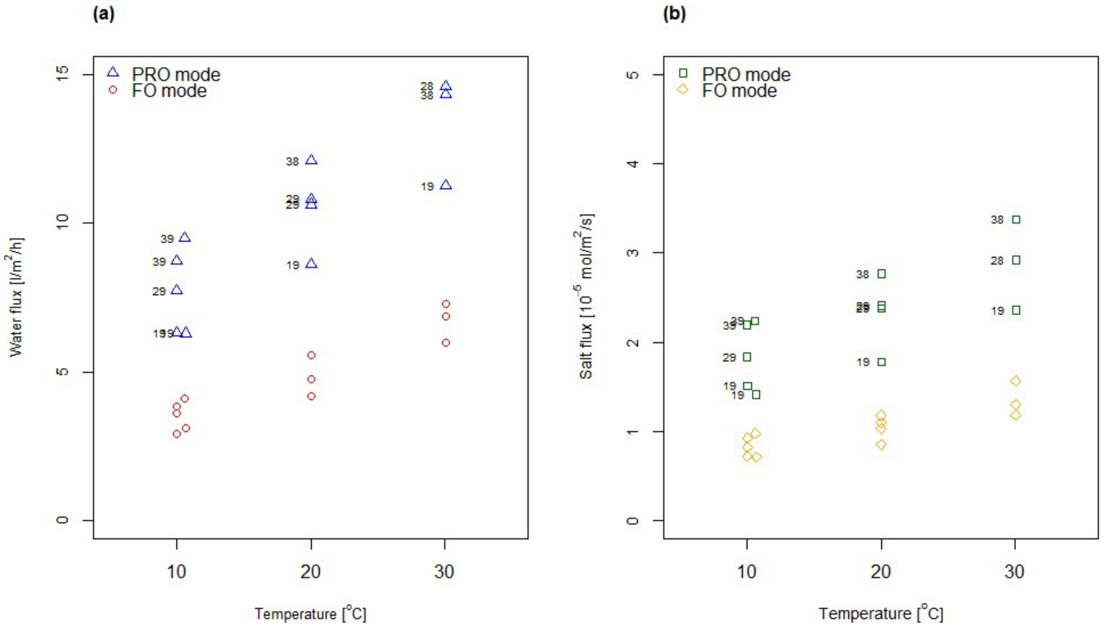

Figure S4. Water (**a**) and salt (**b**) fluxes measured for the TFC3 membrane at different concentrations and temperatures according to a CCD. The figures indicated for each data point (PRO mode only) correspond to the applied draw concentration (g/l NaCl) in that experiment.

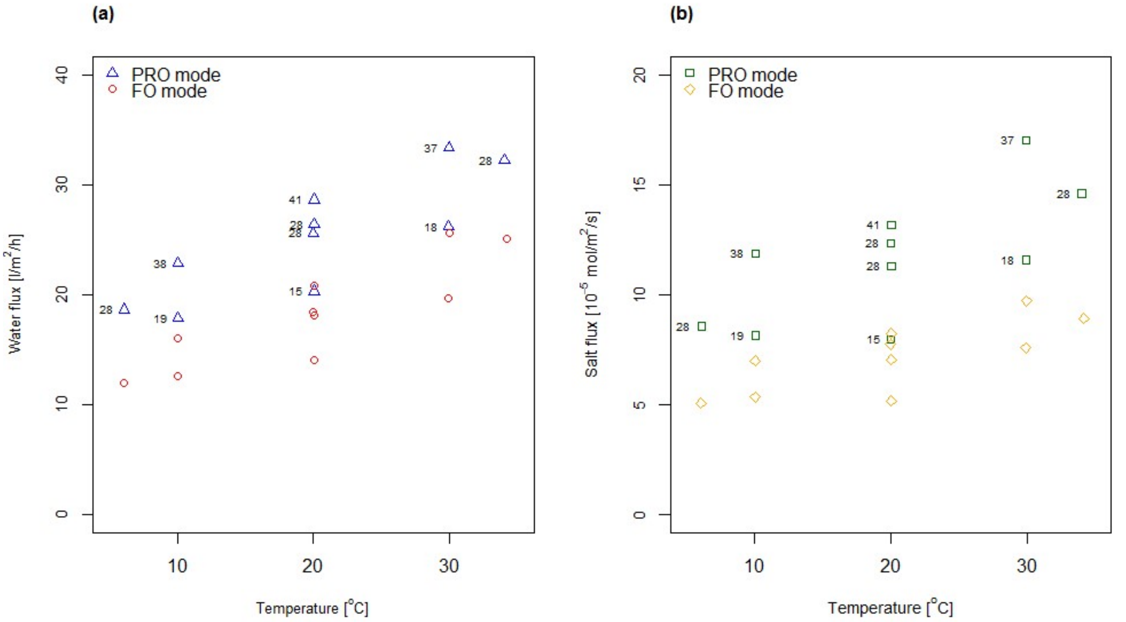

Figure S5.Water (**a**) and salt (**b**) fluxes measured for the TFC4 membrane at different concentrations and temperatures according to a CCD. The figures indicated for each data point (PRO mode only) correspond to the applied draw concentration (g/l NaCl) in that experiment.

3.2 Determination of the Membrane Parameters A, B and S

The separate sets of flux data that was presented in the previous section, each set corresponding to one combination of concentration and temperature, was comprised by two water fluxes and two salt fluxes. Since the ratio of water and salt flux can be considered constant for measurements performed with the same membrane[6], the four fluxes comprise three degrees of freedom that were

utilized to determine the model parameters, *A*, *B* and *S* by applying the transport model described in Section 2.1.

3.2.1 Film Thicknesses

To determine A, B and S by solving the transport model the thicknesses of the boundary layers on the membrane surfaces will need to be determined separately. As shown by Equation (S16) the film thickness will vary according to changes in temperature and concentration due to changes in the Reynolds number and Schmidt number, i.e. changes in diffusion coefficient, density and viscosity. The parameters *b* and *c* in Equation (S14) was set to 0.57 and 0.40, respectively, when evaluating a PRO process [7]. These values have also been used when applying Equation (S16) to determine the relative variation in film thickness with respect to temperature. The relative changes in film thickness as a function of temperature are shown in Figure S6. It can be observed that the relative film thickness decreases with increasing temperature. Further, a sensitivity analysis of changes in the empirical parameters *b* and *c* in Equation (S16) was performed by varying the parameters by ±20 % in the temperature range 5–35 °C. The results show that the largest change in film thickness occurred at 5°C, where an increase in film thickness around 40 % was observed. However, for the remaining temperature range the change in film thickness was less than 20%. This corresponds to a maximum change in the diffusion path of 16 μm, and for most cases typically less than 8 μm. Further, the change in film thicknesses due to changes in concentration according to Equation (S16), i.e. changes in density, viscosity and diffusion coefficient due to changes in concentration, was shown to be less than ± 0.5 % within the concentration range studied.

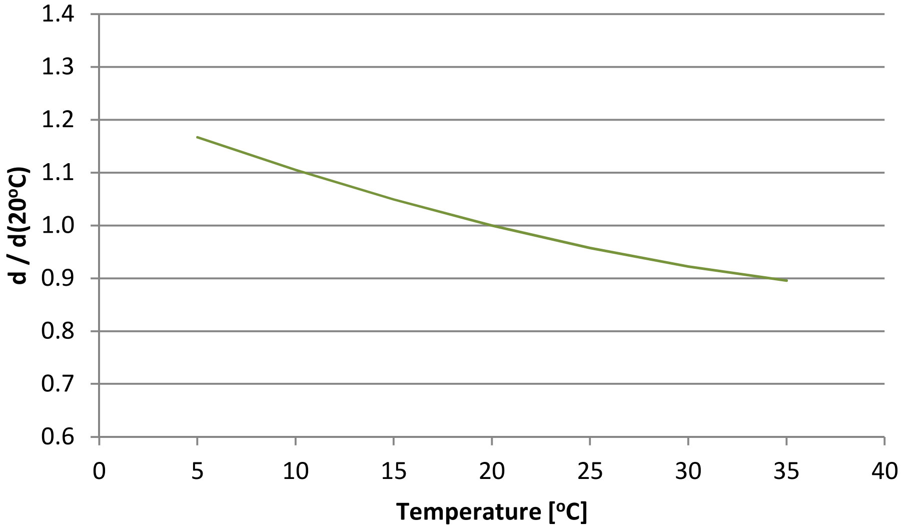

Figure S6. Relative changes in film thickness as function of temperature according to Equation (S16).

Based on the above discussion it was substantiated that the overall resistance for mass transport will be dominated by the structure parameter, and any changes in the film thicknesses due to concentration or temperature will be insignificant compared to the transport resistance related to the structure parameter. Further, the impact of changes in film thickness due to changes in concentration and temperatures were additionally found to be smaller than the uncertainty in the estimation of the structure parameter that were estimated to be around 5–20 % based upon comparison of the results performed at standard conditions for each membrane. Thus, it was decided to keep the film thicknesses constant during the following data analyses.

Thorsen and Holt [1] presented a two-dimensional model describing the mass transport in the diffusion films on each side of the membrane and compared the resulting concentration polarization profile with the film model. They found that a film thickness in the range of 30-50 μm resulted in

conformity between the two models. Thus, a film thickness of 40 µm has been applied for experiments performed according to the standard test protocol.

3.2.2 Structure Parameter

When keeping the film thicknesses constant, the transport model will comprise three unknown parameters, *i.e*. the water permeability (*A*), the salt permeability (*B*) and the structure parameter (*S*). Since the water and salt fluxes have been measured in both FO and PRO mode, the degrees of freedom are sufficient to enable determination of the three parameters at each test condition. Figure S7 shows the impact of variation in concentration and temperature on relative changes in the structure parameter for each of the five different membranes. The average of the replicates determined at the centre point condition has been applied as reference value for each membrane.

Only a minor variation in the structure parameter for the CTA and TFC4 membrane was observed due to changes in temperature and concentration. The membranes TFC2 and TFC3 were also observed to have small variation in the structure parameter, with one or two observations that deviates significantly from unity. On the other hand, a large variation with temperature and concentration was observed in the modelled structure parameter for the TFC1 membrane. The large variation was a result of high uncertainty in the measured salt fluxes for this membrane, which also influenced the modelling for this membrane. This was also observed when analyzing the data with the empirical model in Section 3.1, where the modelled salt flux of TFC1 had the lowest R^2(adj) (cf. Table S2).

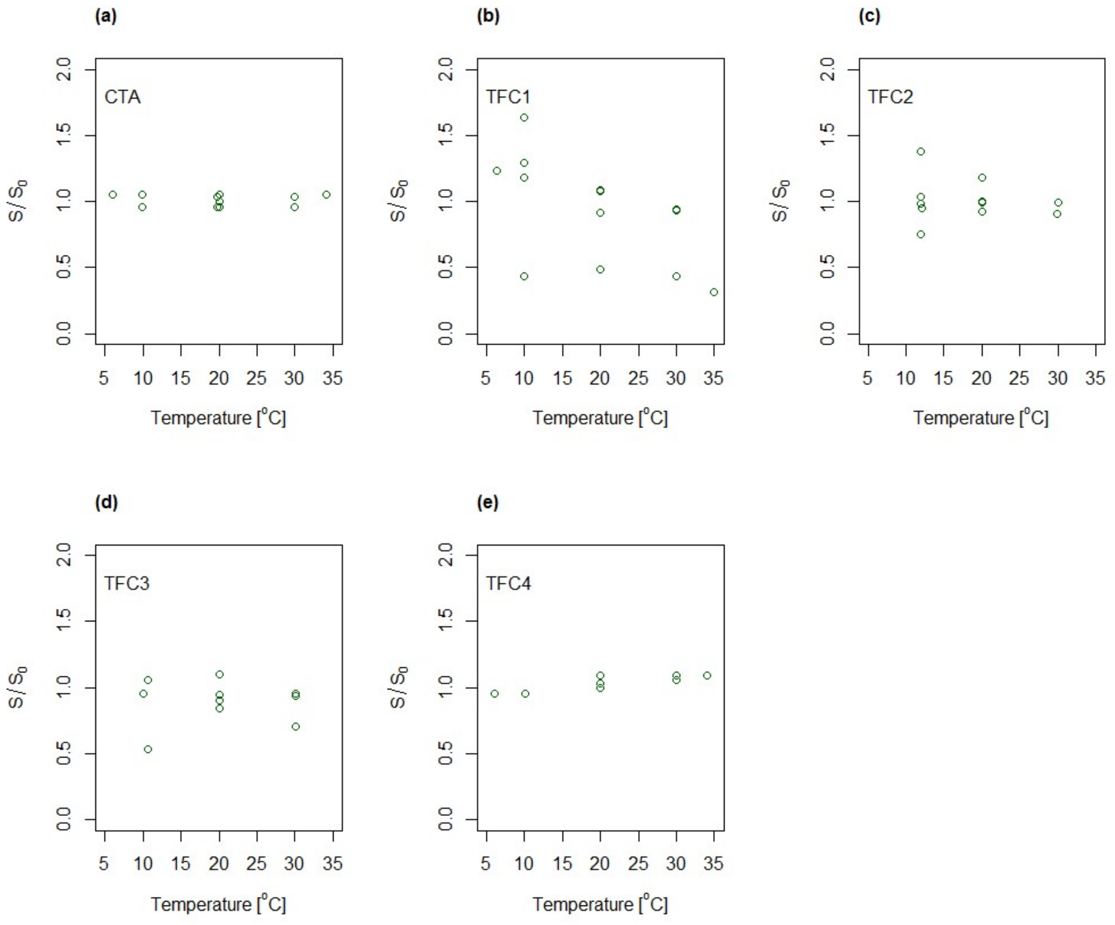

Figure S7. Relative changes in the structure parameter as function of temperature and concentration for (**a**) CTA; (**b**) TFC1; (**c**) TFC2; (**d**) TFC3 and (**e**) TFC4.

To obtain an objective measure of the significance of the observed variation in the modelled structure parameter, an analysis of variance was performed, using the standardized structure parameter, *i.e.* subtracted the mean and divided by the square root of the standard deviation of the structure parameter for each membrane. The p-value for each coefficient, the resulting degrees of freedom from the F-test, including the residual standard error for each membrane are shown in Table S3. None of the coefficients turned out significant at the 5 % level, supporting the hypothesis that the structure parameter is constant within the concentration and temperature range tested.

Table S3. Results from ANOVA of structure parameter listing the p-values for β-coefficients, p-values of the model, the resulting degrees of freedom from the F-test, and the residual standard error from the analysis of variance for each membrane.

	p-value β_0	p-value β_T	p-value β_c	p-value β_{Txc}	p-value model	df	s_{res}
CTA	0.231	0.784	0.234	0.817	0.081	7	0.759
TFC1	0.189	0.093	0.383	0.206	0.102	9	0.831
TFC2	0.187	0.647	0.166	0.639	0.071	7	0.745
TFC3	0.079	0.112	0.065	0.093	0.278	8	0.934
TFC4	0.266	0.119	0.971	0.588	0.007	6	0.483

Table S4. Regression coefficients and R^2(adj) for the relative permeability models developed according to Equation (S13) and Equation (S14), respectively.

	β_A	R^2(adj)	β_B	R^2(adj)
CTA	1.02	0.995	1.01	0.995
TFC1	0.90	0.962	0.80	0.875
TFC2	1.09	0.937	1.05	0.938
TFC3	1.06	0.993	1.04	0.996
TFC4	1.01	0.987	1.00	0.982

References

1. Thorsen, T.; Holt, T. The potential for power production from salinity gradients by pressure retarded osmosis. *Journal of Membrane Science* **2009**, *335*, 103–110.
2. Zhao, S.F.; Zou, L.D. Effects of working temperature on separation performance, membrane scaling and cleaning in forward osmosis desalination (vol 278, pg 157, 2011). *Desalination* **2012**, *284*, 353–353.
3. She, Q.H.; Jin, X.; Tang, C.Y.Y. Osmotic power production from salinity gradient resource by pressure retarded osmosis: Effects of operating conditions and reverse solute diffusion. *Journal of Membrane Science* **2012**, *401*, 262–273.
4. Kim, Y.C.; Elimelech, M. Potential of osmotic power generation by pressure retarded osmosis using seawater as feed solution: Analysis and experiments. *Journal of Membrane Science* **2013**, *429*, 330–337.
5. Mulder, M. *Basic principles of membrane technology*. Kluwer Academic Publishers, Netherlands: 1996.
6. Sivertsen, E.; Holt, T.; Thelin, W.; Brekke, G. Modelling mass transport in hollow fibre membranes used for pressure retarded osmosis. *Journal of Membrane Science* **2012**, *417–418*, 69–79.
7. Achilli, A.; Cath, T.Y.; Childress, A.E. Power generation with pressure retarded osmosis: An experimental and theoretical investigation. *Journal of Membrane Science* **2009**, *343*, 42–52.
8. Myers, R.H.; Montgomery, D.C. *Response surface methodology*. Wiley: New York, 2002; Vol. 2nd ed.

© 2018 by the authors. Submitted for possible open access publication under the terms and conditions of the Creative Commons Attribution (CC BY) license (http://creativecommons.org/licenses/by/4.0/).
